# French-Canadian adaptation and validation of the adolescents’ Pain Coping Questionnaire

**DOI:** 10.1080/24740527.2026.2650298

**Published:** 2026-04-16

**Authors:** Jean Théroux, Laurence Lessard, Stanley Innes, Julien Gardner, Gabrielle Gilbert, Christine Genest, Kate St-Arneault, Janick Carmel, Stéphany Cara-Slavich, Caroline Larue, Stéfan Parent, Soraya Barchi, Isabelle Turgeon, Sylvie LeMay

**Affiliations:** a CHU Sainte-Justine Azrieli Research Center, Montreal, QC, Canada; b Department of Psychology, Université de Montréal, Montréal, QC, Canada; c Department of Mental Health and Wellbeing Program, Eastern Health, South Australia, Australia; d Department of Eastern Health Clinical School, Monash University, Melbourne, Australia; e Faculty of Medicine, Université de Montréal, Montréal, QC, Canada; f Faculty of Nursing, Université de Montréal, Montreal, QC, Canada; g Department of Orthopedic Surgery, Faculty of Medicine, Université de Montréal, Montreal, QC, Canada; h Orthopedic Division, Sainte-Justine University Hospital, Montreal, QC, Canada

**Keywords:** Chronic musculoskeletal pain, scale development and validation, adolescent, coping strategies, psychometric

## Abstract

**Background:**

Musculoskeletal pain affects one in four children, making it the fourth leading cause of disability among youth. The Pain Coping Questionnaire (PCQ) is a well-established instrument for assessing pain-related coping responses; however, no validated French-Canadian version currently exists.

**Aims:**

This research sought to adapt and validate a French-Canadian PCQ (PCQ-F) version for adolescents with musculoskeletal conditions.

**Methods:**

The PCQ was translated into French and reviewed for clarity before its administration to adolescents (ages 10–20) experiencing significant musculoskeletal pain. Validity was evaluated through exploratory factor analysis and its correlation with the Child Post-Traumatic Stress Reaction Index (CPTS-RI), and reliability was assessed using Cronbach’s alpha.

**Results:**

The adaptation and validation of a French-Canadian version of the PCQ involved 176 participants and resulted in a 35-item instrument with seven first-order scales. Behavioral and Cognitive Distraction, Externalizing, Seeking Social Support, and Internalizing/Catastrophizing showed significant (*p* < 0.05) but weak correlation (*r_s_* ≤ 0.36) with the CPTS-RI. The factorial structure explained 65.40% of the variance and demonstrated good to excellent internal consistency for both first- and second-order scales (α ≥ 0.608).

**Conclusion:**

This study addresses the lack of a validated French-Canadian version of the PCQ. Through a cross-cultural adaptation and validation, the resulting PCQ-F demonstrates similar psychometric properties and a factor structure largely consistent with the original instrument. The PCQ-F offers a valid and reliable tool for assessing pain-related coping responses and may support both clinical assessment and future research aimed at promoting adaptive coping and reducing maladaptive cognitive and behavioral responses to pain.

## Résumé

**Contexte**: La douleur musculosquelettique affecte un adolescent sur quatre et représente la quatrième cause d’incapacité chez les jeunes. Le « Pain Coping Questionnaire » (PCQ) est un instrument développé pour évaluer l’adaptation à la douleur. Toutefois, aucune version canadienne-française n’existe actuellement.

**Objectifs**: Cette étude avait pour but d’adapter et de valider une version canadienne-française du PCQ (PCQ-F) chez les adolescents.

**Méthodes**: Le PCQ a été traduit en français et adapté avant son administration chez des adolescents âgés de 10 à 20 ans souffrant de douleurs musculosquelettiques. Des analyses factorielles exploratoires et corrélationnelles (Child Post-traumatic Stress Index) ont évalué sa validité. La fiabilité fut évaluée avec l’alpha de Cronbach.

**Résultats**: Il y a eu 176 participants qui ont répondu au PCQ-F. L’instrument de 35 questions présente une structure à sept facteurs. Les sous-échelles « Behavioral and Cognitive Distraction », « Externalizing », « Seeking Social Support »et « Internalizing/Catastrophizing » ont chacune démontré une corrélation faible (*r_s_* ≤ 0,36) mais significative avec le CPTS-RI (*p* < 0,05). La structure factorielle explique 65,4% de la variance et démontre une cohérence interne ≥ 0,608 pour les échelles de premier et second ordre (α).

**Conclusions**: Cette étude a permis la validation du PCQ-F. Ce dernier présente des propriétés psychométriques semblables à l’instrument original. Le PCQ-F est un outil valide et fiable pour l’évaluation des réponses et des stratégies d’adaptation à la douleur chez les adolescents.

## Introduction

Musculoskeletal pain affects one in four children.^1–3^ As the fourth leading cause of years lived with disability among children and adolescents,^4^ musculoskeletal pain significantly impacts physical activity and schoolwork in 25% of affected adolescents.^5^ Musculoskeletal pain often follows a recurrent, long-term pattern of pain episodes, with a previous episode being the strongest predictor of subsequent pain.^6^ Consistent with international data, approximately one in five children and adolescents in Canada, including Québec, experience chronic pain, most commonly related to headache and musculoskeletal conditions, with higher rates among girls and older adolescents, representing over 110,000 adolescents in Québec alone and highlighting a significant public health concern.^7–9^ Given that the presence of chronic pain in adolescence increases the risk of experiencing chronic pain in adulthood,^10^ understanding how adolescent patients can take action to reduce their pain levels is important for identifying protective and modifiable factors.

General coping strategies are sets of responses to stress that individuals have at their disposal and can use effectively.^11,12^ Unfortunately, not all responses to stress or pain are adaptive. Adaptive coping strategies involve direct and concrete actions aimed at reducing the effects of a stressful event, such as active problem-solving or seeking social support.^13–15^ In contrast, maladaptive responses, such as catastrophizing (a cognitive distortion), avoidance, or behavioral disengagement, may provide short-term relief but are associated with poorer adjustment and worst long-term outcome.^16,17^

Catastrophizing involves rumination and magnification of pain-related threats, while avoidance reflects efforts to distance oneself from a stressor,^18,19^ and behavioral disengagement is characterized by a reduced effort to manage the stressor.^20^ These maladaptive responses are consistently linked to poorer adjustment in adolescents coping with chronic pain,^21,22^ including greater pain interference, emotional distress,^23,24^ and functional limitations.^21,24^ Developmental factors may further amplify these effects during adolescence. Although coping strategies were initially conceptualized within the broader stress literature, their role in pain management has been examined for several decades. Research involving individuals with acute and chronic pain has demonstrated that active, problem-focused coping strategies are generally associated with better functional outcomes, whereas emotion-focused and avoidant responses are linked to greater pain-related disability and distress.^25–27^

Our literature review identified only two pain coping strategy instruments for a pediatric population, namely, the Pain Coping Questionnaire (PCQ) and the Pediatric Pain Coping Inventory (PPCI).^28,29^ Considering that the PPCI had already been translated into French,^30^ we opted to adapt and translate the PCQ for an adolescent population. The development of chronic pain in adolescents following acute traumatic events is well documented. Following a traumatic brain injury, Slaberg et al.^31^ and Tham et al.^32^ reported that nearly 20% and 24.3% of adolescents experienced persistent pain. Furthermore, Groenewald et al. found that adolescents exposed to a greater number (more than four) of adverse childhood experiences, such as parental divorce or witnessing violence, had a 2.7-fold risk of developing chronic pain compared to nonexposed adolescents.^33^ Given these risks, the assessment of pain-related coping responses among French-speaking adolescent is particularly important.

The PCQ is a self-reported measure of pain-related coping responses, encompassing both adaptive coping strategies and maladaptive cognitive and behavioral responses to pain.^29^ It consists of 39 pain coping strategies grouped into eight first-order scales, which are further categorized into three second-order scales: Approach (information seeking, problem-solving, and seeking social support), problem-focused avoidance (positive self-statements, cognitive distraction, and behavioral distraction), and emotion-focused avoidance (externalizing and internalizing/catastrophizing). These three second-order PCQ subscales correspond to distinct clusters of coping strategies that align with the broader coping literature. The approach dimension^34^ reflects active engagement with pain-related stressors and is generally considered adaptive and associated with lower pain-related disability and better emotional adjustment. Problem-focused avoidance^35^ encompasses attention-diverting strategies such as distraction and positive self-statements; these strategies may be helpful in the short term but have shown mixed associations with longer-term pain and functional outcomes. In contrast, emotion-focused avoidance^36–39^ includes strategies aimed primarily at regulating emotional distress, such as catastrophizing and externalizing, which have consistently been linked to higher pain intensity, greater emotional distress, and poorer coping effectiveness. Beyond the original validation in healthy youth and adolescents with juvenile idiopathic arthritis, the PCQ and its subscales have been examined in diverse pediatric populations, including adolescents with musculoskeletal injuries^40,41^ and chronic pain,^41–43^ as well as across multiple language adaptations,^40–44^ supporting its conceptual robustness across clinical and cultural contexts.

Using a 5-point Likert scale (1 = *never* to 5 = *very often*), participants indicate how often they used these coping strategies in response to the prompt, “When I am hurt or in pain for a few hours or days, I … .” The questionnaire mean scores are calculated for each subscale, with higher scores indicating more frequent use of the pain coping strategies measured by the subscale. Two studies conducted by Reid et al. involving children and adolescents aged 8 to 18 validated the questionnaire.^29^ The first was conducted with a healthy sample (*n* = 314) and the second with children with juvenile arthritis (*n* = 28). The authors reported good internal consistency for the eight first-order scales, ranging from α = 0.78 to 0.86, along with suitable construct and predictive validity.^29^

As mentioned above, the PCQ has been translated and validated in several languages including Danish,^44^ Polish,^40^ Finnish,^41^ Catalan,^43^ and Dutch.^42^ However, no validated French-Canadian version currently exists.

Accordingly, the aim of this study was to perform the cross-cultural adaptation and psychometric validation of the French-Canadian version (PCQ-F) in adolescents experiencing musculoskeletal pain. Specifically, we sought to evaluate the factorial structure, reliability, and construct validity of the PCQ-F in this population. We hypothesized that adolescents reporting higher pain intensity, greater emotional distress, and lower perceived pain control would demonstrate higher endorsement of maladaptive pain-related responses and lower use of adaptive coping strategies. This study was approved by the Ethics Committee of the CHU Sainte-Justine (2013-402, 3441).

## Methods

### Translation and content validation procedures

The cross-cultural translation and adaptation of the PCQ followed a standard forward-backward translation process^45^ and was performed between 2016 and 2017. Each step of the translation and validation process involved native French-Canadian–speaking experts and patients from Québec to ensure that it captured the linguistic and sociolinguistic nuances of Québec French.^46^

#### Forward and backward French translation procedure

Initially, a health-related linguist translated the original English version of the PCQ into French. This version was then reviewed by two bilingual, native French-speaking health professionals from the orthopedic clinic at the study setting. Subsequently, two independent clinician nurses back-translated it into English. Afterward, two bilingual experts, an anesthesiologist and an orthopedist, with expertise in pain and musculoskeletal issues reviewed the back-translated version and compared it to the original English text.

#### Expert panel discussions

The final version was submitted to a panel of experts for discussion to address discrepancies and refine the final Québec French-Canadian version. This panel consisted of ten pediatric pain experts (two psychologists, five nurses, and three orthopedic surgeons) who had worked in a pediatric unit at a children’s hospital in Montreal, Canada, for at least 2 years and were fluent in both written and spoken Québec French as well as four patients who had experienced an acute traumatic musculoskeletal event. A research nurse approached the clinicians and patients, explained the research project, and obtained their consent and assent to participate.

#### Content validation process

Each item’s content was evaluated using a self-administered relevance and clarity questionnaire on a Likert scale (1 = *not at all relevant*, 4 = *very relevant*). Following the initial distribution to experts and patients, some items were modified to enhance their comprehension and clarity. This updated version was sent back to four experts for reassessment. As a result, two items were removed from the PCQ-F (item 5, “Go and play” and item 6, “Forget the whole thing”) due to their similarity in Québec French to another item (item 13, “Do something fun” and item 22, “Try to forget it”). The final version of the PCQ-F, comprising 37 items, was approved by the first expert panel and used in the validation process (Table 1).

**Table 1.** Original English and French translated items.

Item	Original items	Items translated to French
1	Ask questions about the problem	Pose des questions à propos de ma douleur
2	Focus on the pain and see how I can make it better	Me concenter sur la douleur et vois comment je peux la diminuer
3	Talk to a friend about how I feel	Parle à un ami de ce que je ressens
4	Tell myself, “Don’t worry everything will be okay”	Me dis ne t’en fais pas, tout ira bien
5	Say mean things to people	Dis des choses méchantes aux gens
6	Worry that I will always be in pain	M’inquiète de toujours ressentir cette douleur
7	Ask a nurse or doctor questions	Pose des questions à un professionnel de la santé (médecin, infirmière, physiothérapeute, etc.)
8	Think of different ways to deal with the problem	Pense à ce que je peux faire pour diminuer ma douleur
9	Talk to someone about how I am feeling	Parle avec quelqu’un de ce que je ressens
10	Say to myself, “Be strong”	Me dis que je dois être courageux
11	Do something fun	Fais quelque chose d’amusant
12	Ignore the situation	Essaie d’ignorer la douleur
13	Argue or fight	Je m’entête et me chicane
14	Keep thinking about how much it hurts	Ne cesse de penser à combien ça me fait mal
15	Find out more information	Recherche plus d’information sur la source de ma douleur
16	Think about what needs to be done to make things better	Pense à différentes façons de faire face à la douleur
17	Tell someone how I feel	Dis à quelqu’un comment je me sens
18	Tell myself it’s not so bad	Me dis que ce n’est pas si pire que ça
19	Do something I enjoy	Fais quelque chose que j’aime
20	Try to forget it	Essaie de l’oublier
21	Yell to let off steam	Crie pour me défouler
22	Think that nothing helps	Pense que rien ne peut m’aider
23	Learn more about how my body works	Apprends davantage comment mon corps fonctionne
24	Figure out what I can do about it	Essaie de trouver des solutions pour soulager ma douleur
25	Talk to a family member about how I feel	Parle à un membre de ma famille de ce que je ressens
26	Say to myself, “Things will be okay”	Me dis que cela va s’améliorer
27	Do something active	Fais une activité physique
28	Don’t think about it	Essai de ne plus penser à ma douleur
29	Get mad and throw or hit something	Me fâche, lance ou frappe des choses
30	Think that the pain will never stop	Pense que la douleur ne s’arrêtera jamais
31	Try different ways to solve the problem until I find one that works	Essaie différentes façons pour soulager la douleur, jusqu’à ce que je trouve celle qui fonctionne
32	Let my feelings out to a friend	Me confie à un ami
33	Tell myself I can handle anything that happens	Me dis que je peux passer au travers de cette situation
34	Do something to take my mind off it	Fais quelque chose pour éviter de penser à la douleur
35	Put it out of my mind	Essaie d’oublier la douleur
36	Curse out loud	Je cris et je dis des gros mots
37	Worry too much about the pain	M’inquiète beaucoup trop de la douleur

#### Sample size and eligibility

Participants were recruited from a pediatric hospital in Montréal. A target sample size of 195 participants was calculated based on the recommended ratio of 5 participants per item for exploratory factor analysis.^47^ The PCQ-F consists of 37 items, which meant that a sample size of 185 participants was adequate. Recruited patients had to meet the following criteria: (1) aged between 11 and 18 years, (2) attending the orthopedic outpatient clinic for a musculoskeletal complaint, and (3) being able to understand, read, and write French. Participants were excluded if they (1) had a cognitive deficit, (2) were diagnosed with a mental health condition or taking psychotropic medications, or (3) were under social care (mentioned in their file). Patients were recruited and screened by the nurse in charge of the orthopedic outpatient clinic during their visit. The research was explained to eligible patients, and consent and assent were obtained from both parents and patients. Participants were invited to complete all the following instruments.

### Instruments

In addition to the translated version of the PCQ (PCQ-F), participants were asked to complete six questionnaires: (1) the Child Post-Traumatic Stress Reaction Index (CPTS-RI), (2) a self-reported numeric rating scale (NRS) for pain, (3) an emotional reaction to pain (Appendix A), (4) an assessment of pain coping effectiveness (Appendix A), (5) a measure of pain and emotion controllability (Appendix A), and (6) a sociodemographic questionnaire. All questionnaires were administered in paper format.

### The Child Post-Traumatic Stress Reaction Index

The CPTS-RI is a questionnaire designed to assess symptoms of posttraumatic stress disorder (PTSD) in children. Initially developed in English,^48^ the questionnaire was translated into French in 2014 and demonstrated good validity and reliability.^49^ The CPTS-RI consists of 20 items where respondents indicate how often they experience PTSD symptoms on a 5-point Likert scale ranging from 0 (*never*) to 4 (*almost always*). The overall score is the sum of the 20 items and falls between 0 and 80, with scores of 12 to 24 considered mild levels of PTSD, 25 to 39 moderate, 40 to 59 severe, and scores over 60 denoting very severe PTSD.^50,51^

### Numeric rating scale

Pain intensity was measured using an NRS. This scale ranges from 0 (*no pain*) to 10 (*worst pain experienced*) and is designed to address three questions that assess changes in pain intensity over time: (1) “What is your current level of pain?,” (2) “What was the highest level of pain you experienced during the past week?,” and (3) “What level of pain did you experience most often during the past week?” The NRS has demonstrated validity and reliability in children and adolescent populations and has been validated in French.^52,53^

The next three questionnaires were used in the initial validation of the PCQ by Reid et al.^29^ and were later employed in other studies that also validated the PCQ. They were used to assess predictive validity. Their psychometric properties were assessed and are detailed in the following sections in the description of each questionnaire. These last three scales were translated and content validated solely on the basis of expert opinion to ensure the translations accurately reflected their original English content. Their detailed description follows.

### Emotional reactions to pain

Participants’ emotional states while experiencing pain were assessed using questions derived from the validation of the original PCQ.^29^ They were asked to rate the intensity of each emotion on a 4-point Likert scale from 0 (*not at all*) to 3 (*very much*). The emotions included happiness, sadness, excitement, anger, calmness, fear, and nervousness. Responses were averaged to provide an overall measure of participants’ emotional distress reactions. In its PCQ validation study, Reid et al. reported an internal consistency of 0.74,^29^ and in a study of children undergoing day surgery, the internal consistency ranged from 0.74 to 0.84.^54^

### Pain coping effectiveness

This questionnaire included seven statements about how participants perceived their ability to manage pain episodes after they concluded. Participants responded using a 5-point Likert scale, ranging from *strongly disagree* to *strongly agree*. The composite score was derived by averaging participants’ scores across all statements. Scores ranged from 1, indicating participants felt they did not handle their pain effectively, to 5, indicating participants felt they managed their pain very effectively. This instrument demonstrated internal consistency of 0.86 and 0.72 in high school and elementary/junior high students, respectively.^55^

### Pain and emotion controllability

This questionnaire included two questions about participants’ beliefs regarding their ability to influence their pain and emotions during painful experiences. Responses were scored using a 5-point Likert scale, from 1 = *never* to 5 = *very often*. The perceived controllability index was calculated by averaging the scores from the questions. A higher score indicated a greater sense of controllability. This instrument has been utilized in other studies^56,57^ beyond Reid et al.^29^ However, despite its consistent use, there is no clear evidence of its independent validation as a standalone instrument.

### Sociodemographic and clinical questionnaires

The sociodemographic and clinical questionnaire collected information on participants’ age, sex, school grade, and the type of pain they were experiencing at the time of completion. This questionnaire was created to best fit the intended purposes of the current study and, as such, did not require validation. This specifically referred to conditions such as backache and not whether the pain was acute or chronic. This multiple-choice question included eight options (Table 2).

**Table 2.** Participants’ sociodemographic and clinical characteristics (*N* = 176).

	n	%	M	SD	Missing	*p*-value
Age	—	—	14.87	2.02	2	
**Sex**			—	—	1	
Female	145	83.33	14.70	2.03		0.003
Male	29	16.67	15.80	1.70		
**Education**					11	
College	21	12.73	—	—		
High school	125	75.76	—	—		
Elementary school	11	6.66	—	—		
Other	8	4.85				
Comorbidities (no)	113	69.75			14	
**Type of pain experienced**^a^					1	
Back pain	160	91.40	—	—		
Muscle pain	67	38.29	—	—		
Headaches	51	29.14	—	—		
Joint pain	36	20.57	—	—		
Menstrual pain	27	20.00	—	—		
Stomachache	23	13.14	—	—		
Earache	8	4.57	—	—		
Other	16	9.14	—	—		
**Pain intensity (0–10)**					7	
Current	—	—	3.31	2.43		
Highest level felt during the week	—	—	6.02	2.42		
Level most often felt during the week	—	—	4.49	2.43		
CPTS-RI (n = 55)						
Mild	21	38.18				
Moderate	25	45.45				
Severe	8	14.54				

^a^Participants may have indicated more than one type of pain.

### Analysis and psychometric assessment

Descriptive statistics, including means and frequencies, were calculated for sociodemographic variables. Reliability was assessed using Cronbach’s alpha.^58^ Construct validity was assessed through factorial analysis, divergent, and predictive validity.^58^ All analyses were performed using IBM SPSS v29 (Armonk, NY: IBM Corp).

### Reliability

Internal consistency was reported for first- and second-order scales using Cronbach’s alphas. A value of 0.7 or greater was considered a good internal consistency, aligning with widely accepted psychometric standards.^59^

## Construct validity

### Factorial analysis

Factorability was confirmed through the interitem correlation matrix, ensuring multiple coefficients greater than 0.3, the Kaiser-Meyer-Olkin (KMO) measure, the anti-image matrix to ensure sampling adequacies, and Bartlett’s test of sphericity, confirming that the correlation matrix was not an identity matrix.^60–62^ Principal axis factoring was used with an oblique rotation due to expected intervariable correlations. The selection of items involved analyzing item loadings, cross-loadings, interitem correlations, floor and ceiling effects, and communality (*h*^2^) values. The higher-order scale was evaluated using the same statistical method as described above.

### Divergent validity

Considering that the PCQ was designed to measure pain-specific coping strategies (cognitive, behavioral, and social responses) rather than psychopathology or overall distress, the CPTS-RI was selected as a theoretically related yet distinct construct to assess divergent validity. Although certain maladaptive coping strategies (e.g., catastrophizing or externalizing) may conceptually relate to emotional distress, the CPTS-RI measures PTSD symptom severity rather than coping responses. Following an assessment of normality, divergent validity was evaluated using Pearson or Spearman correlations by comparing scores on the first-order scales of the PCQ-F and the CPTS-RI. A correlation coefficient less than 0.39 was considered weak, and values between 0.40 and 0.69 or greater than 0.7 were considered moderate or strong, respectively.^63^ We hypothesized that the CPTS-RI score would show weak correlations with the PCQ-F first-order scale reflecting adaptive coping responses and relatively stronger correlations with maladaptive response patterns such as internalizing/catastrophizing or externalizing.^64^

### Predictive validity

A priori hypotheses were examined regarding the PCQ-F subscales and relevant participant characteristics and clinical variables, including sex, age, pain level (NRS), emotional state, and perceived pain control. These hypotheses were grounded in theoretical expectations and prior empirical findings regarding pain coping mechanisms. Statistical analyses employed included linear regression models to assess predictive relationships while controlling for potential confounders (age, sex), *t*-tests and analyses of variance for group comparisons, and Pearson/Spearman correlation analyses to examine associations between continuous variables. Significance was determined at *p* values <0.05.

## Results

### Participants’ sociodemographic and clinical characteristics

Participant recruitment for the validation process occurred between 2017 and 2019. A total of 183 participants were approached, of whom 176 completed PCQs and were included in the analysis. Participants’ sociodemographic and clinical characteristics are detailed in Table 2. The mean age of the sample was 14.87 years (SD 2.02), with boys significantly older (*p* = 0.003). Most participants (*n* = 145, 83.33%) identified as female. Most were attending high school (*n* = 125, 75.76%). Though most participants did not report any comorbidities (*n* = 113, 69.75%), the most common comorbidity noted was musculoskeletal disorders, such as muscular dystrophy (*n* = 18, 12%). Back pain was the most commonly reported pain type (*n* = 160, 91.43%), followed by muscle pain (*n* = 67, 38.29%) and headaches (*n* = 51, 29.14%). Participants were able to report more than one pain type. The mean pain levels experienced in the last week for both the most frequent and most severe pain were 4.49 (SD 2.43) and 6.02 (SD 2.42), respectively. The CPTS-RI was completed by 55 participants, reporting either mild (38.18%) or moderate (45.45%) PTSD symptoms.^48,50^

### Reliability

Internal consistency metrics^65^ for all first- and second-order factors exceeded 0.608 (Tables 3 and 4).

**Table 3.** PCQ-F internal consistency and first-order scales mean scores.

	PS/IS	PSS	CD	Ext	SSS	IC	BD
Internal consistency	0.847	0.777	0.875	0.870	0.907	0.864	0.613
Mean score (SD)	2.89 (0.84)	2.96 (0.85)	3.51 (0.94)	1.39 (0.68)	2.65(1.04)	2.28 (0.99)	2.73 (0.79)
SEM	0.063	0.064	0.071	0.051	0.078	0.075	0.075
% Floor	2.27	1.70	1.14	52.27	8.52	11.36	1.70
% Ceiling	0.57	0.57	7.39	0.00	1.70	1.14	0.00

PS/IS = Problem-Solving/Information Seeking; PSS = Positive Self-Statement; CD = Cognitive Distraction; Ext = Externalizing; SSS = Seeking Social Support; IC = Internalizing/Catastrophizing; BD = Behavioral Distraction.

**Table 4.** Second-order factor analysis results.

	Approach	Emotion-focused Avoidance	Problem-focused Avoidance
Seeking social support	0.851		
Problem solving/Information seeking	0.513		
Internalizing/catastrophizing		0.928	
Externalizing		0.489	
Cognitive distraction			0.853
Positive self-statement			0.511
Behavioral distraction			0.453
Internal consistency	0.637	0.608	0.641

KMO = 0.589; Bartlett’s test < 0.001.

## Construct validity

### Factor analysis

Data from 176 participants were included in the principal axis factoring analysis, using an oblique rotation (oblimin) for both first- and second-order evaluations. The preestablished criteria for factorability were met for the first-order extraction. The correlation matrix displayed several coefficients above 0.3, and visual inspection of the anti-image matrix revealed no items with values below 0.5. The KMO measure was 0.809, and Bartlett’s test was significant (*p* < 0.001), indicating suitability for factor analysis.

We initially analyzed an eight-factor solution to determine whether the French adaptation was comparable to the original extraction by Reid et al.^29^ (Appendix B). This initial extraction of the PCQ-F displayed a pattern similar to the original PCQ. However, two items (item 34, “Do something to take my mind off it,” and item 27, “Do something active”) had issues. Item 34 cross-loaded on two factors, cognitive distraction and internalizing/catastrophizing, and item 27 displayed a low *h*^2^ (0.260) and loaded onto the information seeking factor instead of behavioral distraction. Based on the accepted minimal difference of 0.2 for cross-loading,^66^ item 34 was removed and the extraction was rerun. The new extraction showed some improvement; however, one factor, behavioral distraction, contained only two items, and item 27 remained problematic with a low *h*^2^. Therefore, the extraction was performed again without item 27, which again resulted in the same factor with only two items (Table 5).

**Table 5.** Factor analysis results of the PCQ-F (35-item, eight-factor extraction).

Questions	IS	PSS	CD	Ext	SSS	IC	BD	PS
7. Pose des questions à un professionnel de la santé (médecin, infirmière, physiothérapeute, etc.)	0.610							
1. Pose des questions à propos de ma douleur	0.507							
15. Recherche plus d’information sur la source de ma douleur	0.489							
23. Apprends davantage comment mon corps fonctionne	0.393							
26. Me dis que cela va s’améliorer		0.646						
4. Me dis ne t’en fais pas, tout ira bien		0.614						
18. Me dis que ce n’est pas si pire que ça		0.573						
33. Me dis que je peux passer au travers de cette situation		0.535						
10. Me dis que je dois être courageux		0.467						
35. Essaie d’oublier la douleur			0.826					
20. Essaie de l’oublier			0.821					
28. Essai de ne plus penser à ma douleur			0.773					
12. Essaie d’ignorer la douleur			0.723					
36. Je cris et je dis des gros mots				0.938				
29. Me fâche, lance ou frappe des choses				0.814				
5. Dis des choses méchantes aux gens				0.692				
21. Crie pour me défouler				0.667				
13. Je m’entête et me chicane				0.623				
3. Parle à un ami de ce que je ressens					0.903			
32. Me confie à un ami					0.831			
17. Dis à quelqu’un comment je me sens					0.763			
9. Parle avec quelqu’un de ce que je ressens					0.745			
25. Parle à un membre de ma famille de ce que je ressens					0.571			
30. Pense que la douleur ne s’arrêtera jamais						0.783		
37. M’inquiète beaucoup trop de la douleur						0.744		
6. M’inquiète de toujours ressentir cette douleur						0.702		
22. Pense que rien ne peut m’aider						0.692		
14. Ne cesse de penser à combien ça me fait mal						0.648		
19. Fais quelque chose que j’aime							0.582	
11. Fais quelque chose d’amusant							0.539	
8. Pense à ce que je peux faire pour diminuer ma douleur								−0.829
2. Me concenter sur la douleur et vois comment je peux la diminuer								−0.803
24. Essaie de trouver des solutions pour soulager ma douleur								−0.641
31. Essaie différentes façons pour soulager la douleur, jusqu’à ce que je trouve celle qui fonctionne								−0.567
16. Pense à différentes façons de faire face à la douleur								−0.340

PS/IS = Problem-Solving/Information Seeking; PSS = Positive Self-Statement; CD = Cognitive Distraction; Ext = Externalizing; SSS = Seeking Social Support; IC = Internalizing/Catastrophizing; BD = Behavioral Distraction.

KMO = 0.814; Bartlett’s test < 0.001; total explained variance = 68.46.

Considering the findings above and in light of prior adaptations,^43,44^ a seven-factor extraction was conducted. This analysis revealed that item 23, “Learn more about how my body works,” did not load on any factor and exhibited a low *h*^2^. Similarly, item 27, “Do something active,” showed an *h*^2^ below the acceptable threshold. Moreover, the extraction process led to the merging of two first-order scales, namely, Information Seeking and Problem-Solving. The removal of item 23 caused item 34, “Do something to take my mind off it,” to cross-load on both Cognitive Distraction and Behavioral Distraction subscales, and item 27 continued to demonstrate a low *h*^2^. Subsequently, item 34 was removed, resulting in a final solution where all factors comprised at least four items (Table 6). The factor loadings ranged from 0.324 to 0.953 (Table 4), with a KMO value of 0.810 and a total variance explained of 65.40%. The final version of the PCQ-F is presented in Appendix C.

**Table 6.** Factor analysis results of the PCQ-F (35-item, seven-factor extraction).

	PS/IS	PSS	CD	Ext	SSS	IC	BD
8. Pense à ce que je peux faire pour diminuer ma douleur	0.806						
31. Essaie différentes façons pour soulager la douleur, jusqu’à ce que je trouve celle qui fonctionne	0.748						
2. Me concenter sur la douleur et vois comment je peux la diminuer	0.710						
24. Essaie de trouver des solutions pour soulager ma douleur	0.692						
16. Pense à différentes façons de faire face à la douleur	0.516						
7. Pose des questions à un professionnel de la santé (médecin, infirmière, physiothérapeute, etc.)	0.515						
15. Recherche plus d’information sur la source de ma douleur	0.390						
4. Me dis ne t’en fais pas, tout ira bien		0.611					
26. Me dis que cela va s’améliorer		0.603					
33. Me dis que je peux passer au travers de cette situation		0.542					
18. Me dis que ce n’est pas si pire que ça		0.503					
10. Me dis que je dois être courageux		0.491					
20. Essaie de l’oublier			0.835				
35. Essaie d’oublier la douleur			0.779				
28. Essai de ne plus penser à ma douleur			0.762				
12. Essaie d’ignorer la douleur			0.721				
36. Je cris et je dis des gros mots				0.953			
29. Me fâche, lance ou frappe des choses				0.812			
5. Dis des choses méchantes aux gens				0.725			
21. Crie pour me défouler				0.632			
13. Je m’entête et me chicane				0.613			
3. Parle à un ami de ce que je ressens					0.916		
32. Me confie à un ami					0.847		
17. Dis à quelqu’un comment je me sens					0.748		
9. Parle avec quelqu’un de ce que je ressens					0.744		
25. Parle à un membre de ma famille de ce que je ressens					0.571		
30. Pense que la douleur ne s’arrêtera jamais						−0.763	
37. M’inquiète beaucoup trop de la douleur						−0.730	
6. M’inquiète de toujours ressentir cette douleur						−0.685	
22. Pense que rien ne peut m’aider						−0.679	
14. Ne cesse de penser à combien ça me fait mal						−0.674	
11. Fais quelque chose d’amusant							0.585
19. Fais quelque chose que j’aime							0.579
1. Pose des questions à propos de ma douleur							0.341
27. Do something active							0.324

PS/IS = Problem-Solving/Information Seeking; PSS = Positive Self-Statement; CD = Cognitive Distraction; Ext = Externalizing; SSS = Seeking Social Support; IC = Internalizing/Catastrophizing; BD = Behavioral Distraction.

KMO = 0.810; Bartlett’s test < 0.001; total explained variance = 65.40%.

Considering the two items excluded during the translation process, the final extract demonstrated a structure comparable to that presented by Reid et al.^29^ across five of the seven factors. The factor labeled behavioral distraction encompassed three of the original five items; specifically, item 34, “Do something to take my mind off it,” was excluded during the analysis phase, whereas item 5, “Go and play,” was deleted during the translation. Additionally, item 1, “Ask a question about the problem,” was reallocated from information seeking to this factor. According to Asefi Rad and Wippert,^67^ asking questions may serve to distract from their discomfort, acting as a cognitive or social diversion rather than a deliberate attempt to gather information to solve the issue. The combined information seeking/problem-solving factor contained seven of the original nine items from the respective factors; however, item 23 was removed during factorial analysis, and item 1 was reassigned to the behavioral distraction factor. Except for the Externalizing subscale, which exhibited a high floor effect^68^ (52.27%), no other subscale demonstrated ceiling or floor effects (Table 3).

Factor analysis for the second-order scales met adequacy criteria. Using a methodology similar to that used for the first-order factors, the seven-factor analysis yielded a three-factor solution with a KMO value of 0.646 and a significant Bartlett’s test (*p* < 0.001; Table 6). This second-order factor extraction closely aligned with the one described by Reid et al.^29^ and accounted for a total variance of 69.73%. However, the factor labeled externalizing presented a low *h*^2^. According to various studies,^69–71^ adolescents’ behavioral norms have changed, and aggressive emotional expression is less accepted in today’s adolescent settings, which may cause teens to underreport these behaviors or display them less openly, possibly explaining the low *h*^2^.

### Divergent validity

The following first-order scales showed weak but statistically significant correlations^63^ with the CPTS-RI: Cognitive Distraction (*r_s_* = 0.31, *p* = 0.019; 95% confidence interval [CI] 0.046–0.541), Externalizing (*r_s_* = 0.334, *p* = 0.013; 95% CI 0.067–0.556), Seeking Social Support (*r_s_* = 0.363, *p* = 0.006; 95% CI 0.067–0.556), and Internalizing/Catastrophizing (*r_s_* = 0.315, *p* = 0.019; 95% CI 0.046–0.541). Finally, all second-order scales displayed weak yet statistically significant correlation with the CPTS-RI (Approach: *r_s_* = 0.38, *p* = 0.004; 95% CI 0.137–0.59; Problem-Focused Avoidance: *r_s_* = 0.35, *p* = 0.008; 95% CI 0.114–0.556; Emotion-Focused Avoidance: *r_s_* = 0.34, *p* = 0.012; 95% CI 0.078–0.556). These weak correlations support the divergent validity of the PCQ-F, indicating that pain coping strategies represent a construct distinct from PTSD severity. Although relatively stronger correlations were hypothesized for maladaptive coping scales, the relatively small number of participants with higher CPTS-RI scores (*n* = 8, 14.54%) may have limited the ability to detect stronger associations.^72^

### Predictive validity

Group differences were analyzed and showed no significant variations with respect to age or sex (*p* > 0.05), even after dichotomizing age using two alternative cut points (10–13 versus 14–20) adolescents. Table 7 outlines the correlations between participants’ pain (current, most severe, and most often reported) and the PCQ-F first- and second-order scales. Several subscales showed moderate (Internalizing/Catastrophizing) and weak correlations (Problem-Solving/Information Seeking, Externalizing, Behavioral Distraction) with current pain, all of which were significant (*p* < 0.05). Participants’ most severe pain also moderately, but significantly, correlated with the Internalizing/Catastrophizing subscale (*p* < 0.001) and had a weaker, yet significant, correlation with Problem-Solving/Information Seeking and Externalizing (*p* < 0.05). Lastly, the most often reported pain showed weak but significant correlation with the PCQ-F first-order scales Problem-Solving/Information Seeking and Internalizing/Catastrophizing (*p* < 0.05). This suggests that participants experiencing more severe pain tended to score higher on these PCQ-F first-order scales, suggesting they used more pain coping strategies. When examining the second-order scales, only Approach and Emotion-Focused Avoidance showed low and moderate but significant correlations, respectively, with the participants’ pain levels (current, most severe, and most often; *p* < 0.05).

**Table 7.** Correlations between PCQ-F first-order scales and participants’ pain levels.

	Current			Most severe			Most often		
Pain level	*r_s_*	*p*	95% CI	*r_s_*	*p*	95% CI	*r_s_*	*p*	95% CI
Subscales									
Problem-Solving/Information Seeking	0.175	**0.022**	0.022–0.321	0.242	**0.001**	0.091–0.382	0.239	**0.002**	0.088–0.380
Positive Self-Statements	−0.023	0.766	−0.177 to 0.132	−0.028	0.719	−0.181 to 0.127	−0.074	0.335	−0.226 to 0.081
Cognitive Distraction	0.084	0.275	−0.071 to 0.235	0.149	0.052	−0.005 to 0.297	0.112	0.145	−0.043 to 0.262
Externalizing	0.196	**0.01**	0.042–0.340	0.195	**0.011**	0.042–0.339	0.103	0.180	−0.052 to 0.253
Seeking Social Support	0.121	0.116	−0.034 to 0.270	0.097	0.207	−0.058 to 0.248	0.027	0.725	−0.128 to 0.181
Internalizing/Catastrophizing	0.414	**< 0.001**	0.277–0.535	0.413	**< 0.001**	0.276–0.533	0.353	**< 0.001**	0.210–0.481
Behavioral Distraction	0.197	**0.01**	0.044–0.341	0.149	0.051	−0.005 to 0.297	0.061	0.427	−0.094 to 0.214
Higher-order scales									
Approach	0.173	**0.024**	0.019–0.319	0.199	**0.009**	0.046–0.343	0.150	**0.50**	−0.004 to 0.298
Emotion-Focused Avoidance	0.410	**< 0.001**	0.273–0.531	0.400	**< 0.001**	0.262–0.522	0.326	**< 0.001**	0.180–0.457
Problem-Focused Avoidance	0.090	0.243	−0.066 to 0.241	0.126	0.102	−0.030 to 0.275	0.032	0.673	−0.123 to 0.186

r_s_: Spearman rho; CI: confidence interval.

Bold represent significant values (*p* < 0.05).

Correlation analysis was also conducted to explore relationships among the PCQ-F first- and second-order scales, participants’ emotion and pain reactiveness and controllability, and pain coping effectiveness (Table 8). Emotion and pain reactiveness showed a positive and significant correlation with the Problem-Solving/Information Seeking, Externalizing, Internalizing/Catastrophizing, and Seeking Social Support subscales (*p* ≤ 0.05). Pain and emotion controllability were significantly and positively correlated with Positive Self-Statement (*p* < 0.001) and negatively with Externalizing and Internalizing/Catastrophizing (*p* ≤ 0.05). Lastly, pain and coping effectiveness were significantly and positively correlated with Positive Self-Statement, Seeking Social Support, and Behavioral Distraction (*p* ≤ 0.05) and negatively correlated with Externalizing and Internalizing/Catastrophizing (*p* ≤ 0.05).

**Table 8.** Correlations between PCQ-F first- and second-order scales and emotion and pain reactiveness and controllability and pain coping effectiveness.

	Emotion and pain reactiveness			Emotion and pain controllability			Pain coping effectiveness		
	*r_s_*	*p*	95% CI	*r_s_*	*p*	95% CI	*r_s_*	*p*	95% CI
Subscales									
Problem-Solving/Information seeking	0.218	**< 0.004**	0.064–0.361	0.105	0.174	−0.051 to 0.256	0.076	0.324	−0.08 to 0.229
Positive Self-Statements	0.059	0.447	−0.097 to 0.212	0.287	**< 0.001**	0.138–0.423	0.297	**< 0.001**	0.149–0.433
Cognitive Distraction	0.003	0.974	−0.153 to 0.158	0.091	0.240	−0.065 to 0.243	0.051	0.512	−0.105 to 0.205
Externalizing	0.443	**< 0.001**	0.309–0.560	−0.200	**0.009**	−0.345–0.047	−0.204	**0.008**	−0.349–0.051
Seeking Social Support	0.167	**0.030**	0.011–0.314	0.079	0.310	−0.078 to 0.231	0.212	**0.006**	−0.059–0.356
Internalizing/Catastrophizing	0.559	**< 0.001**	0.442–0.657	−0.404	**< 0.001**	−0.527–0.266	−0.357	**< 0.001**	−0.485–0.213
Behavioral Distraction	0.010	0.894	−0.145 to 0.165	0.150	0.052	−0.006 to 0.298	0.236	**0.002**	0.084–0.378
Higher-order scales									
Approach	0.212	**0.006**	0.059–0.356	0.094	0.224	−0.062 to 0.246	0.185	**0.016**	0.031–0.331
Emotion-Focused Avoidance	0.600	**< 0.001**	0.491–0.691	−0.362	**< 0.001**	−0.490–0.219	−0.348	**< 0.001**	−0.478–0.204
Problem-Focused Avoidance	0.029	0.707	−0.127 to 0.184	0.224	**0.003**	0.071–0.367	0.264	**< 0.001**	0.114–0.403

r_s_: Spearman rho; CI: confidence interval.

Second-order scales Approach and Problem-Focused Avoidance demonstrated positive, albeit modest, statistically significant correlations with emotion reactivity to pain and pain coping effectiveness (*p* < 0.05) and with perceived controllability and pain coping effectiveness (*p* < 0.05). Conversely, Emotion-Focused Avoidance exhibited a strong positive correlation with emotion reactivity to pain (*p* < 0.001) and a significant negative correlation with perceived controllability and pain coping effectiveness (*p* < 0.001).

The relationship between the PCQ first- and second-order scales and the participants’ emotional reaction to pain, perceived controllability, and pain coping effectiveness was examined using a regression analysis, adjusting for sex and age (regression results are reported in Appendix D).

The regression model revealed that the PCQ first-order scales collectively explained a significant portion of the variance in participants’ emotional reactions to pain, perceived controllability, and pain coping effectiveness. All models were statistically significant (*p* < 0.001) and accounted for approximately 35.3%, 25.5%, and 27.6% (adjusted *R*^2^) of the variance in pain and emotional reactivity, pain and emotional controllability, and pain coping effectiveness, respectively. When examining the PCQ-F second-order scales, all models remained statistically significant (*p* < 0.001) and explained between 22.37% and 34.32% (adjusted *R*^2^) of participants’ variance.

Moreover, the following PCQ first-order scales emerged as significant factors contributing to emotional reactions to pain, perceived controllability, and coping effectiveness, respectively: Externalizing and Internalizing/Catastrophizing (*p* (HC3) < 0.05) for emotional reaction to pain; Internalizing/Catastrophizing (*p* heteroskedasticity (HC3) < 0.001) for perceived controllability, and Internalizing/Catastrophizing, Seeking Social Support, and Behavioral Distraction (*p* (HC3) < 0.05) for pain coping effectiveness. Participants who exhibited stronger emotional reactions to pain generally scored higher on PCQ’s emotion-focused components, such as externalizing and internalizing/catastrophizing, and also tended to perceive less control over their pain. Those who demonstrated good pain coping effectiveness more frequently employed strategies such as seeking social support and behavioral distraction.

## Discussion

This study details the cultural adaptation and validation of the PCQ into French Canadian. The adapted instrument comprises 35 items organized into seven subscales, with each subscale demonstrating a reliability coefficient above 0.8, except for the Behavioral Distraction subscale, which exhibited an internal consistency of 0.613. The PCQ-F demonstrated satisfactory content validity and reliability. Consistent with our hypotheses, the subscale coping scores showed weak correlations with CPTS-RI scores, supporting the instrument’s divergent validity and confirming its suitability as a targeted measure of pain coping strategies.

Two items were deleted during the adaptation process based on our preestablished statistical and conceptual criteria, resulting in a more robust factor structure. Additionally, the original Information Seeking and Problem-Solving subscales identified by Reid et al.^29^ merged into a single factor in the present analysis. Despite these modifications, the extracted structure largely mirrored that of the original instrument, with five of the seven first-order factors aligning directly with those reported by Reid et al.^29^ This consistency across versions supports the cross-cultural stability of the PCQ’s core constructs.

Like other cross-cultural PCQ adaptations, items were removed due to statistical or translation issues. For example, in the Danish adaptation,^44^ three items were removed (items 1, 9, and 36), a decision later supported by Huguet et al.^43^ for the Catalan adaptation. Despite its eight-factor solution, Marttinen et al.^41^ also deleted one item (item 27) due to its poor fit, resulting in a 38-item Finnish-adapted questionnaire. Furthermore, Bąk et al.,^40^ who adapted the PCQ into Polish, examined only the PCQ’s second-order structure for both children and parents. Despite this, they also removed one item (item 5) that failed to load on the parent adaptation. Item deletion is not unusual during the cross-cultural adaptation of questionnaires, especially when they exhibit poor cultural relevance, unclear meaning, or problematic measurement properties.^45,73,74^ Indeed, behaviors considered acceptable or salient in the 1990s may no longer reflect contemporary adolescents’ experiences, potentially influencing item endorsement patterns and factor structure.^75^

Similar to the Danish^44^ and Catalan^41^ adaptations, items from the Information Seeking and Problem-Solving first-order scales merged to form a single subscale. These scales’ strategies represent proactive approaches to pain management, identifying problematic components through information seeking and testing potential solutions via problem-solving, thereby linking items across both subscales. Furthermore, these subscales are incorporated under a higher-order construct, namely, “approach,” which reinforces their interconnectedness.^29,38,40^ Thastum et al.’s^44^ combined factor Information Seeking/Problem-Solving contains seven items, six of which matched our subscales. The missing item (item 23), “Learn more about how my body works,” was deleted during the factorial analysis because it failed to load on any factor. Removal of this item is unique to the French adaptation, likely due to cultural or linguistic context, because it may have been perceived as vague or less meaningful for French adolescents.^45^

Though not all patients exposed to a traumatic event will develop psychopathology, some may experience internalized symptoms, such as acute PTSD, or externalized symptoms, like behavioral problems and aggressiveness.^76–78^ Despite some conflicting evidence,^79^ coping seems to be directly and positively correlated with PTSD,^80–83^ and assessing specific coping strategies is likely more informative than relying on a global rating of coping.^82^

Patients with PTSD symptoms generally report higher pain levels,^84^ suggesting they utilize fewer pain coping strategies. Consistent with expectations for divergent validity, correlations between CPTS-RI scores and PCQ-F coping scales were weak. This pattern indicates that pain-related coping strategies represent a construct distinct from PTSD symptom severity. Although pain coping strategies and PTSD are both related to stress, they are conceptually and empirically distinct.^85^ Despite the complexity of the relationship between coping strategies and PTSD, studies have shown that maladaptive strategies such as internalizing/catastrophizing and externalizing, often associated with higher pain intensity, are linked to greater levels of PTSD symptoms,^64,86^ as demonstrated in our study, where these maladaptive strategies showed a small to moderate but significant correlation with the participants’ CPTS-RI questionnaire. However, other research has shown conflicting results regarding the relationship between adaptive coping and PTSD symptoms.^87–89^

Our study demonstrated a positive yet low to moderate correlation with current pain levels and four PCQ-F first-order scales (Problem-Solving/Information Seeking, Externalizing, Internalizing/Catastrophizing, and Behavioral Distraction). This relationship aligns with findings from Reid et al.,^29^ who reported that participants with high pain were more likely to adopt emotion-focused avoidance strategies such as externalizing and internalizing/catastrophizing. Though Thastum used experimental pain in patients with juvenile arthritis, internalizing strategies were the most utilized.^44^ Considered a maladaptive emotion-avoidant pain coping strategy, externalizing and internalizing/catastrophizing have been associated with higher levels of pain.^90,91^ Interestingly, contrary to other studies,^29,43,44^ problem-solving/information seeking showed a positive correlation with participants’ pain. This association was also reported by Marttinen et al.^41^ and Bandell-Hoekstra et al.^42^ Although problem-solving is generally regarded as an adaptive coping strategy, it may be linked to higher pain intensity or to participants with chronic pain, for whom problem-solving coping is not as effective.^92^

Emotional reactivity can be defined as an individual’s emotional response to an event, such as pain or changes in the environment.^93^ Adolescents with heightened reactivity tend to demonstrate stronger responsiveness to environmental influences.^94,95^ According to Jensen et al., individuals with greater emotional reactivity to pain often resort to more maladaptive coping strategies, such as catastrophizing.^96^ Our findings support this, because emotional reactivity to pain was correlated with internalizing/catastrophizing. Previous studies that validated the PCQ also reported similar results.^29,40,43^

In our study, participants’ perceptions of control and coping effectiveness were inversely correlated with the use of maladaptive strategies. A greater perception of control over pain and coping effectiveness is associated with numerous positive outcomes for patients with chronic pain.^97^ As a result, patients who feel more in control and are better at coping with pain tend to use fewer maladaptive and more problem-focused strategies, such as positive self-statement, as reported by Reid et al.^29^ and Bak et al.^40^

## Strengths and limitations

Though the study of pain coping strategies mainly relates to individuals experiencing significant acute or chronic pain, the original PCQ and several translations primarily included healthy children and adolescents. Our research is notable as one of the few that specifically recruited participants coping with trauma-related acute or chronic pain.

There are several limitations to our study. One notable limitation is the absence of a temporal reliability assessment for the instrument, which could reveal sources of error unrelated to the instrument itself. Because participants were recruited from a single pediatric hospital in Montréal, this may limit the generalizability of the findings to other regions or settings. Although cognitive or mental health impairment was not a concern for this study and this exclusion was methodologically justified, it may limit the applicability of the PCQ-F to a more diverse clinical population. Another limitation is the lack of validation for younger children (under 10 years old). The original PCQ was developed and validated for children and adolescents aged 8 to 18 years. However, we aimed to collect clinical data specifically on adolescents (10–20 years) with musculoskeletal injuries. Considering that the selected pain coping strategies may be influenced by both the participant’s pain intensity and age, the combined effects of these factors may have impacted the factor structure of the PCQ-F in different ways.^98,99^ Therefore, the questionnaire requires further evaluation in a younger population.

Another potential explanation for why our factor structure differed from the original PCQ could be related to the participants’ mean age (14.85 years) and the number of female participants (84%), because participants in the Reid et al.^29^ validation study were younger (12.7 years) and had a more balanced proportion of females (56%). Through cognitive, developmental, and sociocultural mechanisms, age and gender can influence the factor structure, leading to items clustering into distinct constructs.^100–103^

We also want to acknowledge the low completion rate for the CPTS-RI questionnaire, which only 55 participants completed. A larger number of completed questionnaires might have strengthened our a priori hypothesis regarding the divergent validity.

Finally, the sample size may have affected the factorial analysis. Despite the lack of consensus, a sample size of five participants per question is recommended.^47^ However, only 176 completed questionnaires were returned and included in the analysis. Similarly, the only study on the adaptation of the PCQ, with a smaller sample, was the Finnish version,^41^ which only had 92 participants, whereas other studies on the adaptation of the PCQ had sample sizes ranging from 220 to 2871 participants.^40,42–44^

## Conclusion

In conclusion, the rigorous validation process followed yielded a version of the PCQ-F that is both culturally and structurally valid in a population of French-Canadian adolescents. This adaptation fills a critical gap in the measurement of pain coping in this population, thus providing a valid and reliable tool to identify specific interventions aimed at enhancing adaptive coping and reducing maladaptive cognitive and behavioral responses to pain, such as internalizing patterns, catastrophizing, and avoidance.

## Future research avenue

Considering the availability of the PCQ short form,^104^ it would be worthwhile to translate and adapt this shorter version to French-Canadian. Additionally, it would be valuable to assess the instrument’s sensitivity to change in response to interventions, as well as its applicability in other French-speaking populations beyond Québec.

## Appendix A

Emotional reactions to pain

## Appendix B

Pattern matrix extraction 37 items, 8 factors

**Table:**

	Factor							
	PS	PSS	CD	Ext	SSS	IS	IC	BD
PCQ_8	0.826							
PCQ_2	0.761							
PCQ_24	0.681							
PCQ_31	0.635							
PCQ_16	0.417							
PCQ_26		0.626						
PCQ_4		0.622						
PCQ_18		0.594						
PCQ_33		0.537						
PCQ_10		0.452						
PCQ_35			0.833					
PCQ_20			0.827					
PCQ_28			0.771					
PCQ_12			0.711					
PCQ_34			0.462				0.301	
PCQ_36				0.942				
PCQ_29				0.816				
PCQ_5				0.678				
PCQ_21				0.675				
PCQ_13				0.606				
PCQ_3					0.896			
PCQ_32					0.831			
PCQ_17					0.774			
PCQ_9					0.763			
PCQ_25					0.589			
PCQ_1						−0.533		
PCQ_7						−0.529		
PCQ_23						−0.434		
PCQ_15						−0.431		
PCQ_27						−0.303		
PCQ_19							0.672	
PCQ_11							0.556	
PCQ_30								0.782
PCQ_37								0.720
PCQ_6								0.697
PCQ_22								0.692
PCQ_14								0.645

PS/IS = Problem-Solving/Information Seeking; PSS = Positive Self-Statement; CD = Cognitive Distraction; EXT = Externalizing; SSS = Seeking Social Support; IC = Internalizing/Catastrophizing; BD = Behavioral Distraction.

## Appendix C

### Questionnaire PCQ-F

Voici des énoncés qui représentent ce que les gens peuvent dire ou faire lorsqu’ils ressentent de la douleur. Nous sommes intéressés par les choses que tu fais ou tu ressens lorsque tu as de la douleur pendant quelques heures ou quelques jours. Pour chaque question, en cercle le chiffre qui montre combien de fois tu effectues les actions mentionnées:

**Table:**

1 = *Jamais*	2 = *Presque jamais*	3 = *Quelques fois*	4 = *Souvent*	5 = *Très souvent*					
LORSQUE JE SUIS SOUFFRANT OU RESSENT DE LA DOULEUR POUR QUELQUES HEURES OU PLUSIEURS JOURS, JE …					*Jamais*	*Presque jamais*	*Parfois*	*Souvent*	*Très souvent*
Pose des questions à propos de ma douleur					1	2	3	4	5
Me concenter sur la douleur et vois comment je peux la diminuer					1	2	3	4	5
Parle à un ami de ce que je ressens					1	2	3	4	5
Me dis, ne t’en fais pas, tout ira bien					1	2	3	4	5
Dis des choses méchantes aux gens					1	2	3	4	5
M’inquiète de toujours ressentir cette douleur					1	2	3	4	5
Pose des questions à un professionnel de la santé (médecin, infirmière, physiothérapeute)					1	2	3	4	5
LORSQUE JE SUIS SOUFFRANT OU RESSENT LA DOULEUR POUR QUELQUES HEURES OU PLUSIEURS JOURS, JE …									
Pense à ce que je peux faire pour diminuer ma douleur					1	2	3	4	5
Parce avec quelqu’un de ce que je ressens					1	2	3	4	5
Me dis que je dois être courageux					1	2	3	4	5
Essaie d’ignorer la douleur					1	2	3	4	5
Je m’entête et me chicane					1	2	3	4	5
Ne cesse de penser à combien ça me fait mal					1	2	3	4	5
Rechercher plus d’informations sur la source de ma douleur					1	2	3	4	5
Pense à différentes façons de faire face à la douleur					1	2	3	4	5
Dis à quelqu’un comment je me sens					1	2	3	4	5
Fais quelque chose que j’aime					1	2	3	4	5
LORSQUE JE SUIS SOUFFRANT OU RESSENT À DOULEUR POUR QUELQUES HEURES OU PLUSIEURS JOURS, JE …									
Me dis que ce n’est pas si pire que ça					1	2	3	4	5
Essaie de l’oublier					1	2	3	4	5
Crie pour me défouler					1	2	3	4	5
Pense que rien ne peut m’aider					1	2	3	4	5
Essaie de trouver des solutions pour soulager ma douleur					1	2	3	4	5
Parle à un membre de ma famille de ce que je ressens					1	2	3	4	5
Fais une activité physique					1	2	3	4	5
Me dis que cela va s’améliorer					1	2	3	4	5
LORSQUE JE SUIS SOUFFRANT OU RESSENT À DOULEUR POUR QUELQUES HEURES OU PLUSIEURS JOURS, JE …									
Essaie de ne plus penser à ma douleur					1	2	3	4	5
Me fâche, lance ou frappe des choses					1	2	3	4	5
Pense que la douleur ne s’arrêtera jamais					1	2	3	4	5
Essaie différences façons pour soulager la douleur, jusqu’à ce que je trouve celle qui fonctionne					1	2	3	4	5
Me confie à un ami					1	2	3	4	5
Me dis que je peux passer au travers de cette situation					1	2	3	4	5
Fais quelque chose pour éviter de penser à la douleur					1	2	3	4	5
Essaie d’oublier la douleur					1	2	3	4	5
Je cris et je dis des gros mots					1	2	3	4	5
M’inquiète beaucoup trop de la douleur					1	2	3	4	5

## Appendix D

**Table D1.** PCQ First-Order Scales Regression Results for Emotion Reaction to Pain.

	B	95% CI for B	SE B	β	R^2^	Adjusted R^2^
Model					0.388	0.353
Constant	0.204	−0.141:0.549	0.175			
Problem-Solving/Information Seeking	0.025	−0.057:0.107	0.042	0.045		
Positive Self-Statement	0.082	−0.001:0.164	0.042	0.148		
Cognitive Distraction	−0.028	−0.101:0.045	0.037	−0.055		
Externalizing	0.184	−0.085:0.282	0.050	0.261*		
Seeking Social Support	0.017	0.052:0.086	0.035	0.037		
Internalizing/Catastrophizing	0.206	0.133:0.278	0.037	**0.428*****		
Behavioral Distraction	−0.078	−0.166:0.010	0.045	−0.131		
Age	0.000	0.000:0.357	0.000	−0.059		
Sex 1 versus 0	0.056	0.214:0.485	0.080	0.119		

B = unstandardized regression coefficient; CI = confidence interval; SE B = standard error of the coefficient; ß = standardized coefficient; R^2^ = coefficient of determination, **p* (HC3) < 0.05; ***p* (HC3) < 0.01; ****p* (HC3) < 0.001.

Bold represent significant values (*p* < 0.05).

**Table D2.** PCQ First-Order Scales Regression Results for Pain and Emotion Controllability

	B	95% CI for B	SE B	β	R^2^	Adjusted R^2^
Model					0.295	0.255
Constant	2.657	2.040:3.273	0.312			
Problem-Solving/Information Seeking	0.122	−0.025:0.269	0.074	0.134		
Positive Self-Statement	0.114	−0.034:0.261	0.075	0.123		
Cognitive Distraction	0.015	−0.115:0.145	0.066	0.018		
Externalizing	−0.014	−0.190:0.162	0.089	−0.012		
Seeking Social Support	0.020	−0.103:0.143	0.062	0.026		
Internalizing/Catastrophizing	−0.373	−0.502:-0.244	0.065	**−0.467*****		
Behavioral Distraction	0.166	0.008:0.324	0.080	0.168		
Age	−0.001	−0.002:0.001	0.001	−0.073		
Sex 1 versus 0	0.061	−0.220:0.343	0.143	0.078		

B = unstandardized regression coefficient; CI = confidence interval; SE B = standard error of the coefficient; ß = standardized coefficient; R^2^ = coefficient of determination, **p* (HC3) < 0.05; ***p* (HC3) < 0.01; ****p* (HC3) < 0.001.

Bold represent significant values (*p* < 0.05).

**Table D3.** PCQ First-Order Scales Regression Results for Pain Coping Effectiveness

	B	95% CI for B	SE B	β	R^2^	Adjusted R^2^
Model					0.314	0.276
Constant	2.869	2.27:3.468	0.303			
Problem-Solving/Information Seeking	−0.015	−0.158:0.128	0.072	−0.017		
Positive Self-Statement	0.072	−0.071:0.215	0.073	0.079		
Behavioral/Cognitive Distraction	−0.020	−0.146:0.106	0.064	−0.025		
Externalizing	−0.085	−0.256:0.086	0.087	−0.074		
Seeking Social Support	0.194	0.074:0.313	0.061	**0.260****		
Internalizing/Catastrophizing	−0.297	−0.423:-0.172	0.063	**−0.378****		
Behavioral Distraction	0.191	0.038:0.344	0.077	**0.196****		
Age	0.002	0.000:0.003	0.001	0.166		
Sex 1 versus 0	0.011	−0.262:0.285	0.138	0.015		

B = unstandardized regression coefficient; CI = confidence interval; SE B = standard error of the coefficient; ß = standardized coefficient; R^2^ = coefficient of determination, **p* (HC3) < 0.05; ***p* (HC3) < 0.01; ****p* (HC3) < 0.001.

Bold represent significant values (*p* < 0.05).

**Table D4.** PCQ Second-Order Scales Regression Results for Emotion Reaction to Pain

	B	95% CI for B	SE B	β	R^2^	Adjusted R^2^
Model					0.363	0.343
Constant	0.223	−0.123:0.569	0.175			
Approach	0.047	−0.031:0.126	0.040	0.082		
Emotion-Focused Avoidance	0.371	0.287:0.456	0.043	**0.559*****		
Problem-Focused Avoidance	−0.024	−0.119:0.070	0.048	−0.034		
Age	−0.001	−0.001:0.000	0.000	−0.089		
Sex 1 versus 0	0.076	−0.080:0.233	0.079	0.162		

B = unstandardized regression coefficient; CI = confidence interval; SE B = standard error of the coefficient; ß = standardized coefficient; R^2^ = coefficient of determination, **p* (HC3) < 0.05; ***p* (HC3) < 0.01; ****p* (HC3) < 0.001.

Bold represent significant values (*p* < 0.05).

**Table D5.** PCQ Second-Order Scales Regression Results Pain and Emotion Controllability

	B	95% CI for B	SE B	β	R^2^	Adjusted R^2^
Model					0.247	0.234
Constant	2.658	2.031:3.284	0.317			
Approach	0.119	−0.023:0.261	0.072	0.124		
Emotion-Focused Avoidance	−0.460	−0.613:-0.307	0.078	−0.416		
Problem-Focused Avoidance	0.302	0.131:0.472	0.086	**0.252*****		
Age	−0.001	−0.002:0.001	0.001	−0.055		
Sex 1 versus 0	0.018	−0.266:0.301	0.143	0.023		

B = unstandardized regression coefficient; CI = confidence interval; SE B = standard error of the coefficient; ß = standardized coefficient; R^2^ = coefficient of determination, **p* (HC3) < 0.05; ***p* (HC3) < 0.01; ****p* (HC3) < 0.001.

Bold represent significant values (*p* < 0.05).

**Table D6.** PCQ Second-Order Scales Regression Results for Pain Coping Effectiveness

	B	95% CI for B	SE B	β	R^2^	Adjusted R^2^
Model					0.264	0.241
Constant	2.799	2.189:3.409	0.309			
Approach	0.231	0.092:0.369	0.070	0.245		
Emotion-Focused Avoidance	−0.443	−0.592:-0.294	0.076	**−0.406*****		
Problem-Focused Avoidance	0.207	0.040:0.373	0.084	**0.175*****		
Age	0.002	0.001:0.003	0.001	0.189		
Sex 1 versus 0	−0.058	−0.333:0.218	0.140	−0.075		

B = unstandardized regression coefficient; CI = confidence interval; SE B = standard error of the coefficient; ß = standardized coefficient; R^2^ = coefficient of determination, **p* (HC3) < 0.05; ***p* (HC3) < 0.01; ****p* (HC3) < 0.001.

Bold represent significant values (*p* < 0.05).
